# Heterogeneous digital biomarker integration out-performs patient self-reports in predicting Parkinson’s disease

**DOI:** 10.1038/s42003-022-03002-x

**Published:** 2022-01-17

**Authors:** Kaiwen Deng, Yueming Li, Hanrui Zhang, Jian Wang, Roger L. Albin, Yuanfang Guan

**Affiliations:** 1 Department of Computational Medicine and Bioinformatics, University of Michigan, Ann Arbor, MI USA; 2 Eli Lilly and Company, Indianapolis, IN USA; 3 Department of Neurology, University of Michigan, Ann Arbor, MI USA; 4 VAAAHS GRECC, Ann Arbor, MI USA; 5 Department of Internal Medicine, University of Michigan, Ann Arbor, MI USA

**Keywords:** Machine learning, Parkinson's disease

## Abstract

Parkinson’s disease (PD) is one of the first diseases where digital biomarkers demonstrated excellent performance in differentiating disease from healthy individuals. However, no study has systematically compared and leveraged multiple types of digital biomarkers to predict PD. Particularly, machine learning works on the fine-motor skills of PD are limited. Here, we developed deep learning methods that achieved an AUC (Area Under the receiver operator characteristic Curve) of 0.933 in identifying PD patients on 6418 individuals using 75048 tapping accelerometer and position records. Performance of tapping is superior to gait/rest and voice-based models obtained from the same benchmark population. Assembling the three models achieved a higher AUC of 0.944. Notably, the models not only correlated strongly to, but also performed better than patient self-reported symptom scores in diagnosing PD. This study demonstrates the complementary predictive power of tapping, gait/rest and voice data and establishes integrative deep learning-based models for identifying PD.

## Abstract (web summary)

Deng et al. develop deep learning methods that identify Parkinson’s Disease (PD) patients using public accelerometer and position data with higher accuracy than when using gait/rest and voice-based models. Their study demonstrates the complementary predictive power of tapping, gait/rest and voice data and establishes integrative deep learning-based models for identifying PD.

## Introduction

Parkinson’s disease (PD) is one of the first disorders for which digital biomarkers were explored. The clinical features of PD include movement abnormalities such as tremors, bradykinesia, and rigidity^1^. Mobile devices and built-in sensors like accelerometers and gyroscopes provide the ability to convert the features into digital signals as quantitative surrogates of PD symptoms. The success of distinguishing the person with Parkinson’s (PwP) from otherwise healthy individuals in the general population using the digital biomarkers will enable a remote and more convenient way for symptom evaluations and diagnosing, with minimal interruptions in the participants’ daily life^2,3^.

A multitude of public datasets is available^4^ for analyzing various types of digital biomarkers such as voice recordings^2,5,6^, movement data^2,7,8^, the magnetic resonance imaging (MRI)^9^, and the handwriting patterns^9,10^. And the application of machine learning or deep learning techniques like Support Vector Machine^11,12^ and the Convolutional Neural Network^13^ successfully build diagnostic models on these biomarkers. For example, a community-based challenge benchmarked algorithms using 30-s rest and gait data from cell phones to differentiate self-reported PwP from healthy subjects^14,15^.

Despite a considerable number of studies using digital biomarkers for PwP detection, prior studies on the movement data have mainly focused on the evaluation of gross motor skills such as walking and rest with medium sample sizes^4^. In addition to gross motor function impairment, PwP typically experiences difficulty in daily tasks requiring fine-motor skills, *e.g*., picking up objects, buttoning, tying shoelaces, and writing^16^. Abnormalities of fine-motor coordination are not only characteristic of PwP, but their presence is often a more sensitive indicator of PwP than changes in gait or balance, particularly in early phase PD. As a result, clinicians often use qualitative analysis of simple fine movements, such as finger tapping, to assess patients for possible parkinsonism. Additionally, assessment of fine movements is a component of standard clinical severity rating scales for PwP research^17^. However, there have been few digital biomarker studies focusing on fine-motor skills, and the applications of state-of-the-field machine learning techniques such as deep learning approaches only report a moderate performance^13^. Instead, prior studies with better performances mostly have focused on traditional signal extraction methods^18–20^. In addition, there are no fair comparisons or integration of algorithms across different types of motor assessments aimed at differentiating PwP from healthy individuals.

In this study, we aim at addressing the above open question with an innovative self-reported dataset collected by mPower^2^. mPower is an APP designed for collecting data from PwP and otherwise healthy individuals, including 20–30-s walking data, 10-s voice data, and 20-s finger tapping data. For a substantial number of individuals (2729), all three data types are available, allowing parallel evaluations of the performance of the models.

We first applied the deep learning algorithms on the finger-tapping accelerometer data. Then we construct the models on the coordinates of the tapping positions, under the expectation that PD patients exhibit worse coordination and slower motion during the tapping task compared to healthy individuals. This will lead to inaccurate and/or changing positions of the tapping contact points. The tapping coordinate models outperformed the gait/rest algorithm and the voice algorithm in differentiating PwP from otherwise healthy individuals. We further integrated all three types of models: finger tapping, voice, and rest/gait, and achieved an AUC of 0.944 in diagnosing PD. Importantly, further evaluations with AUCs on the individuals with self-reported UPDRS (The Unified Parkinson’s Disease Rating Scale) scores show that our model can significantly outperform the symptom scores. The AUC of our work is 0.949, and the AUCs of UPDRS scores are 0.823, and 0.935 when using the UPDRS motor experience part.

## Results

The goal of this study is to explore and integrate digital biomarkers of movement beyond the gross motor skills captured by gait and rest evaluations. We will present the development of the models for tapping records, followed by outlines of models for voice and walking data. The latter have benchmarks established by previous studies^15,21,22^. Finally, we will present the performance of the integrated models and demonstrate the clinical utility of the models by comparing with patient self-reports.

### Model built on accelerometer data of tapping performs well in identifying PD

**Fig. 1:**
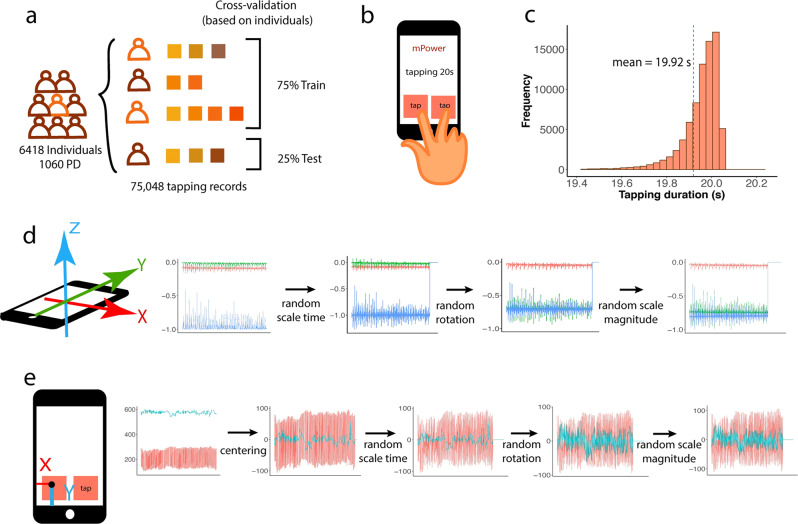
Summary of tapping data and preprocessing techniques. **a** Training and test data are separated by individuals to avoid contamination. **b** Participants were asked to tap successively in turns on the screen of the phone for 20 s. Machine learning models are trained for accelerometer and coordinate data separately. **c** Distribution of the length of the records (**d**, **e**) demonstrate the preprocessing and augmentation techniques for raw accelerometer and coordinate data. Normalization/centering, random time scaling, rotation, and magnitude scaling were applied in a sequential order. At the last step, magnitude scaling was applied to each channel of the accelerometer and coordinated data.

The tapping data was obtained from the mPower portal ([https://www.synapse.org/#!Synapse:syn5511439/tables/]). Data included a total of 8,003 individuals. Among them, 6418 have self-reported diagnosis, and 1060 are self-identified as having a professional diagnosis of PD (Table 1). We obtained a total of 78,879 records (75048 of them have the self-reported diagnosis), ranging from 1 to 522 for each individual (the median record number is 3; 75% of them have less than 6 records, and 95% of them have less than 30). On average, the individuals with PD have 40.2 records, and the individuals without PD have 6.06. Because each individual has multiple records, individual level, instead of record-level cross-validation was carried out in all analyses to prevent overestimation of the performance^23,24^. Specifically, in each round of cross-validation, 75% of the individuals and all their records are used as the training set, and the remaining 25% of individuals are used as the test set (Fig. 1a).

**Table 1:** Demographics of the individuals used in the tapping study.

Demographic type	Demographic value	Individual number of PD	Individual number of Non-PD	Total individual number
Gender	Male	698 (13.9%)	4329 (86.1%)	5027
	Female	359 (26.1%)	1014 (73.9%)	1373
	Prefer not to answer	0 (0.0%)	6 (100.0%)	6
	(Missing data)	3	9	12
Age	≤35	36 (0.95%)	3753 (99.04%)	3789
	35–50	158 (13.3%)	1034 (86.7%)	1192
	50–65	509 (56.2%)	397 (43.8%)	906
	>65	352 (71.5%)	140 (28.5%)	492
	(Missing data)	5	34	39
Smoke	No	696 (17.4%)	3299 (82.6%)	3995
	Yes	362 (16.6%)	1820 (83.4%)	2182
	(Missing data)	2	239	241

During the tapping task, participants were asked to alternately tap two fingers of the dominant hand on the touchscreen of the phone, placed flat on a table, within two contiguous squares (Fig. 1b). Participants were asked to tap as quickly as possible for 20 s. Nearly 99% of the 75048 actual collected data have the duration times located between 19.4 and 20.2 s. The average duration time is 19.92 s, with a standard deviation 0.481 (Fig. 1c). Two types of data are recorded. The first type is the accelerometer, composed of [x, y, z] coordinate values sampled at 100 Hz (Fig. 1d). The second type is the location of the tapping on the screen, recorded as [*x*, *y*] coordinate values (Fig. 1e).

**Fig. 2:**
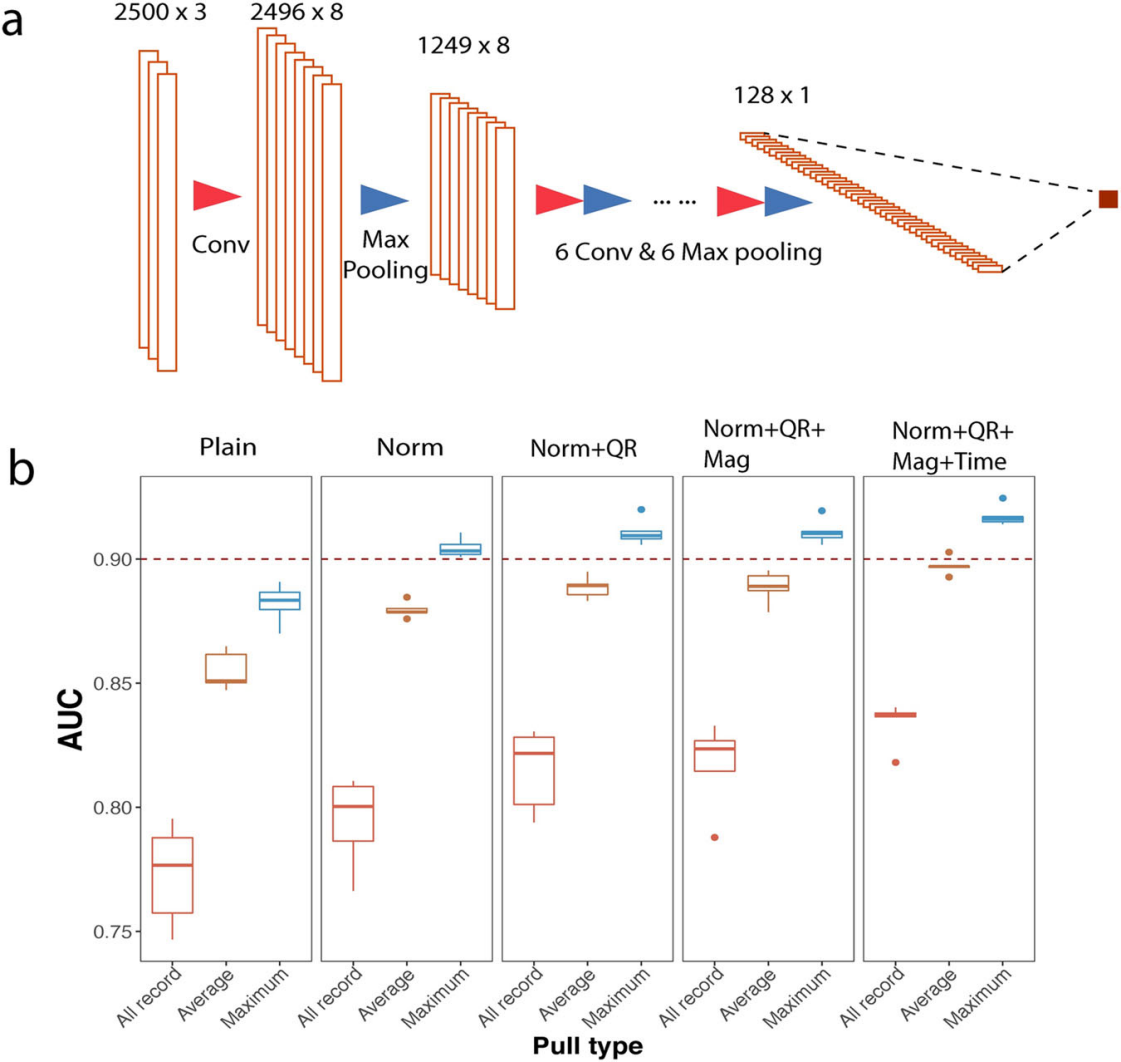
Augmentation and normalization improve the performance of the tapping accelerometer model. **a** represents the model structure for the accelerometer data tapping model with seven convolutional layers, seven max-pooling layers, and 1 fully connected layer. **b** “Plain Model” represents the model using raw data without augmentation or normalization. “Norm” represents the model with Z-score normalization. “Quaternion Rotation” (QR), “Magnitude”, and “Time” denote the three types of augmentation methods. The data were pre-processed in sequential order of the methods shown above the plot. “All record” indicates the performance evaluated on the record level; “Average” and “Maximum” represent the individual level performance by using the average or the maximum prediction of all records of the same individual. The models with normalization and all augmentation methods showed the top performance.

We first developed models that use accelerometer data to predict PD. When participants tap on the screen, the tapping motion induces acceleration of the phone. Thus, accelerometer data is capable of capturing the magnitude, direction and speed for the movement of the phone. A 1D deep learning network architecture was implemented, where the only dimension is time. [x, y, z] values were used as three channels of the input, analogous to color channels in image analysis (Fig. 2a). Timewise perturbation, magnitude augmentation, and spatial augmentation by rotating the record reference frames progressively improved model performance (Fig. 2b, Supplementary Table 1). As each individual had multiple records, we took the maximal or average prediction values across all records for each individual. Taking the maximal values appeared to perform better than taking the average of all records, as it ensures the PD individuals always have as high scores as possible, and generates a good separation with the non-PD individual scores (Fig. 2b). This likely reflects fluctuations in motor performance in PwP with the maximal values capturing peak abnormalities that are most predictive of PD. The performance (mean AUC value) from 5-fold cross-validation is 0.8340 at the record level, and 0.9174 at the individual level.

### Deep learning models based on tapping coordinate data predict PD accurately

The mPower tapping coordinate data is composed of *x*, *y* positions on the cell phone screen, and the timestamp of each tap. The average number of taps in a single record is 153, ranging from 2 to 359. Before the deep learning models, we made exploratory experiments on the machine learning techniques using the time-series data feature extraction algorithms. We extracted 1508 features for each record using the Python package tsfresh^25^, and fit a LightGBM model^26^. The dataset split and pulling methods are the same as those in the deep learning models. The average AUC is 0.692, and the performances are highly dependent on the training and test data, ranging from 0.6 to 0.92 in the 5-fold cross-validation.

**Fig. 3:**
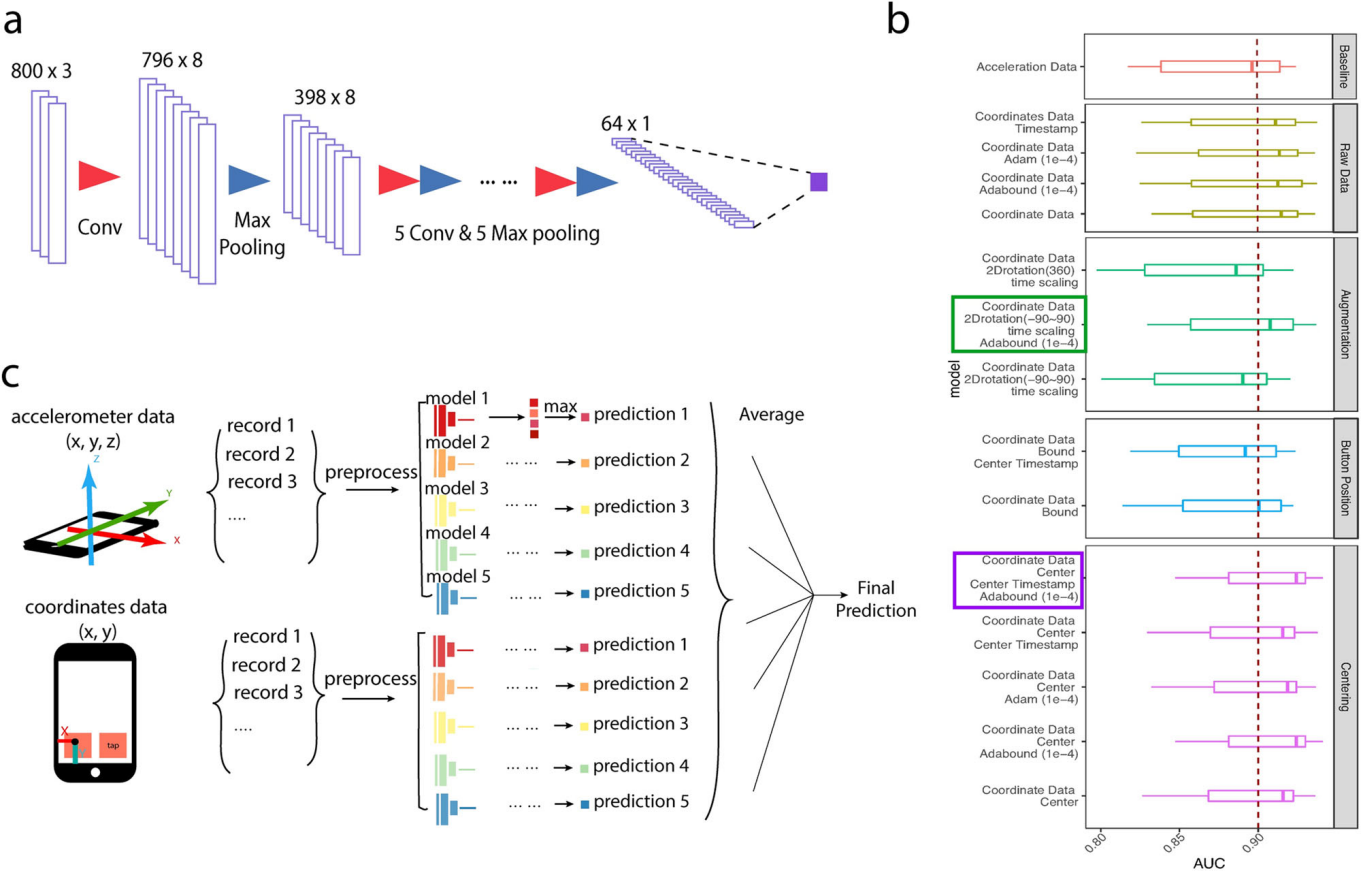
Model design for tapping coordinate data. **a** depicts the model structure for the coordinate data tapping model (inputs: *x*, *y* coordinates and timestamp). The model contains six convolutional layers, six max-pooling layers, and one fully connected layer. **b** Experiments were designed to search for the optimal data processing methods and network hyperparameters, including the normalization strategy on the coordinates and the timestamps, augmentations with 2D-rotation and time scale, and the network optimizer. Best performing models by augmentation and centering groups are enclosed in squares. **c** A combined model is generated by averaging the prediction scores from accelerometer and from coordinates.

We imported these records into a 1D deep learning network. We tested a variety of models including training models with or without adding the timestamps (Supplementary Table 2; Supplementary Fig. 1A), applying diverse augmentation and normalization methods (Supplementary Table 3, 4; Supplementary Fig. 1B, 2), and tuning the models with a series of hyperparameter settings (Supplementary Table 2; Supplementary Fig. 1A). We found that centering timestamp and coordinate data, and using Adabound improved model performance (Fig. 3b). On the other hand, other techniques, including normalizing the coordinates by subtracting the button position and 2D-rotation with a random angle in a specific range (−90° to 90° and 0°–360°), impaired performances (Fig. 3b, Supplementary Fig. 2). During cross-validation, the AUC was 0.9352, compared to 0.9174 using accelerometer data (*p* < 0.05 for the cross-validation performances*)*.

### Comparison of tapping models with voice and gait/rest models for predicting PD

In order to compare the performance of the tapping models and the fine-motor skills, voice, and gait and rest (accelerometer) models for predicting PD. We identified a total of 2729 individuals (645 PwP) in the mPower dataset who had all three types of data. We retested the above accelerometer and coordinate models based on the tapping records of this set of individuals by cross-validation.

**Fig. 4:**
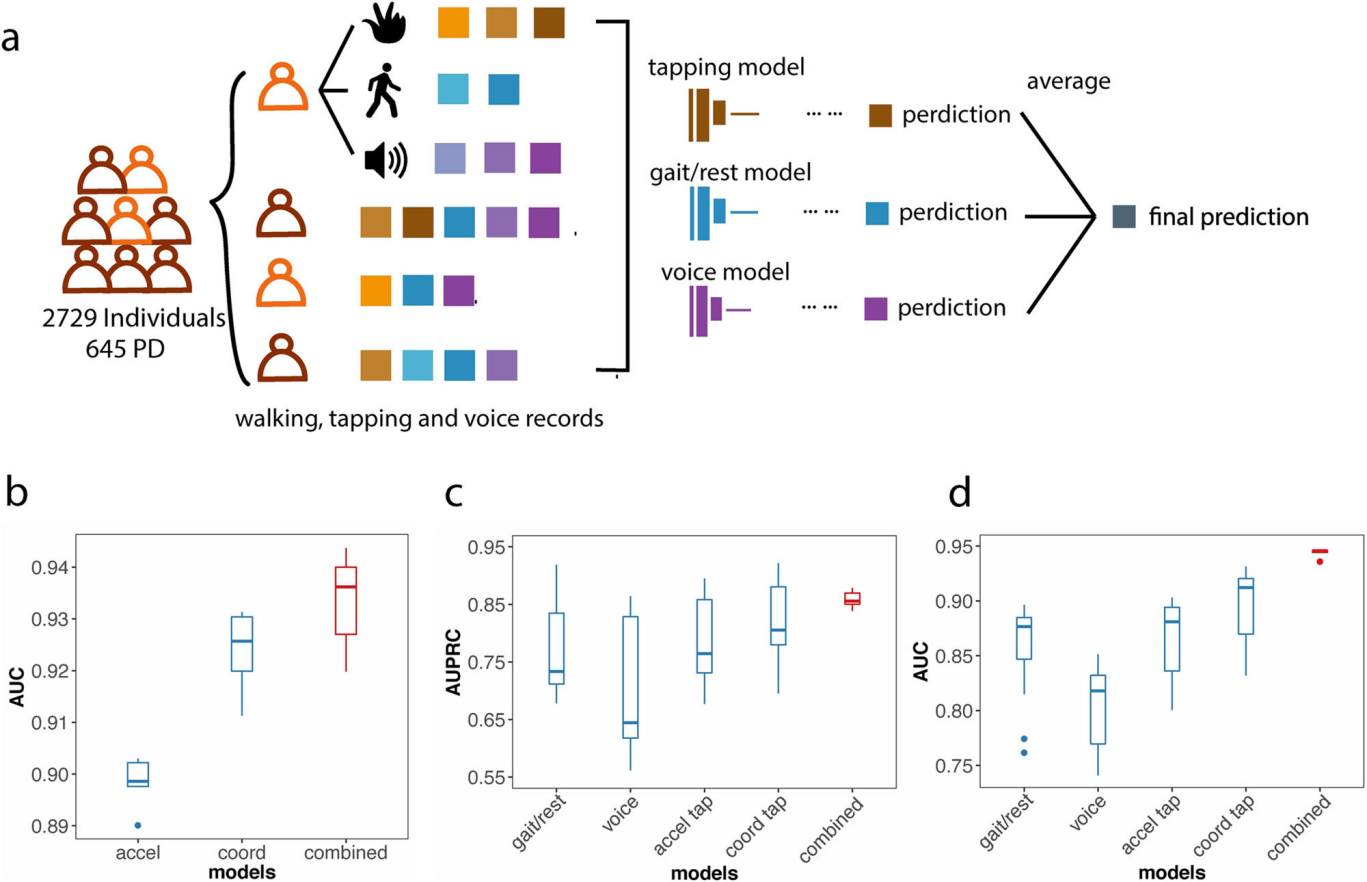
Combining tapping, voice and gait/rest data improves performance in diagnosing PD. **a** A total of 2729 individuals (645 PD) who had all three types of data were split into the same cross-validation folds and to train gait/rest, voice, and tapping models. The models are combined together by averaging the prediction scores from each model. **b** Assembling accelerometer and coordinate tapping data models exceeds the performance of either single model. (**c**) and (**d**) depict the comparison among gait/rest, voice, tapping, and assembled models. The combined model achieves the best performance.

Next, we retrained a model for gait and rest for this set of individuals. This model follows Zhang et al.^15^, which was a top-performing model in the DREAM Parkinson’s disease challenge. Briefly, the accelerometer data of the cell phone during 20- or 30-s walking and rest activities were input into a 1D deep learning network of three channels, integrating spatial and time augmentation (Fig. 4). The models were trained separately for walking and rest. Then, we took the maximal value of the predictions across all records. The walking data achieved an average AUC of 0.8983.

Additionally, we trained a voice model, using a 1D deep learning network of one channel. The voice data in the mPower dataset are audio recordings of the participants saying ‘Ahh..’ for 10 s, sampled at a frequency of 44.1 kHz. We tested various modeling techniques and found that time-wise and magnitude-wise augmentation could improve the performance (Supplementary Fig. 3). The voice data achieved an average AUC of 0.8335 at the individual level in this population.

We also trained the accelerometer and coordinate tapping models with this population, which achieved average AUCs of 0.8983 and 0.9236. We assembled the two models by averaging the prediction scores from the two machine learning models for the same individual and predicted PwP status with the assembled score (Fig. 3c). The assembled model produces better performance than either of the single models, with an average AUC of 0.9333 (Fig. 4b). We found that tapping models significantly outperformed voice and gait/rest models in this population (*p* < 0.05 for both of the cross-validation performances, Fig. 4d).

We examined whether assembling the voice and gait/rest models in conjunction with tapping data models can further improve predictive performance (Fig. 4a). After averaging the evaluation scores from the four models (walk, voice, accelerometer tapping, and coordinate tapping) and using the assembled score to predict disease status, the average AUC reached 0.944, better than any single model (*p* < 0.05 for the cross-validation performances, Fig. 4d). This suggests that the evaluation scores of different models may have specific limitations and that assembling all sources of information may alleviate the individual limitations and obtain better performance (Fig. 4c, d).

### Robust performance across demographic groups and comparison to patient self-reports

**Fig. 5:**
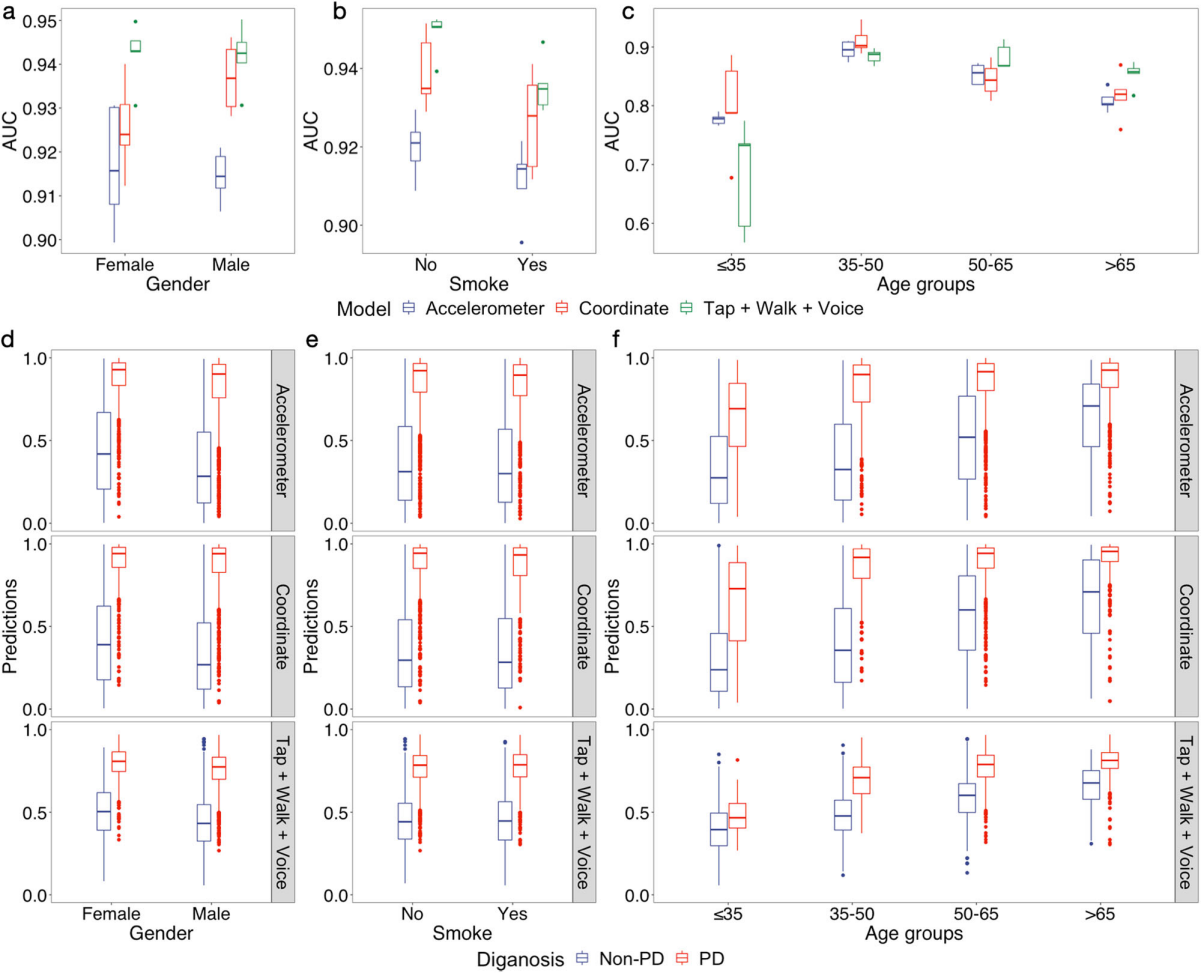
Performance comparison of different demographic groups. **a**–**c** Performances in AUC and the predictions in different groups of data, separated by gender, smoking and age. **d**–**f** Distribution of prediction scores of Non-PD and PD for different gender, smoking, and age groups.

Previous studies of Parkinson’s Disease presented the relationships between the risk, symptom and the demographics, such as the advancing age^27,28^, gender^29,30^, and the smoking behavior^31,32^. We examined if the performance is robust against these diverse demographic groups. We found that the performance remains similar and strong for different genders, smoking groups, and age groups, other than the age group < = 35 years old (Fig. 5a–c). All of the AUCs of the age groups older than 35 are higher than 0.85. We also evaluate our assembling model on the individuals older than 45 (the average individual number is 520), and have an average AUC of 0.885, 0.25 higher than the previous work on the same age group^13^. Age group ≤35 has an average AUC of 0.8 in the tapping models, and 0.681 in the assembling models. Of note, only 0.8% of this population has a positive PD diagnosis. Additionally, the prediction values of PD and normal groups are well separated into different demographic groups (Fig. 5d–f). As age increases, the overall prediction values increase as expected, due to more PD patients. Prediction values are independent of smoking status (Supplementary Fig. 4).

**Fig. 6:**
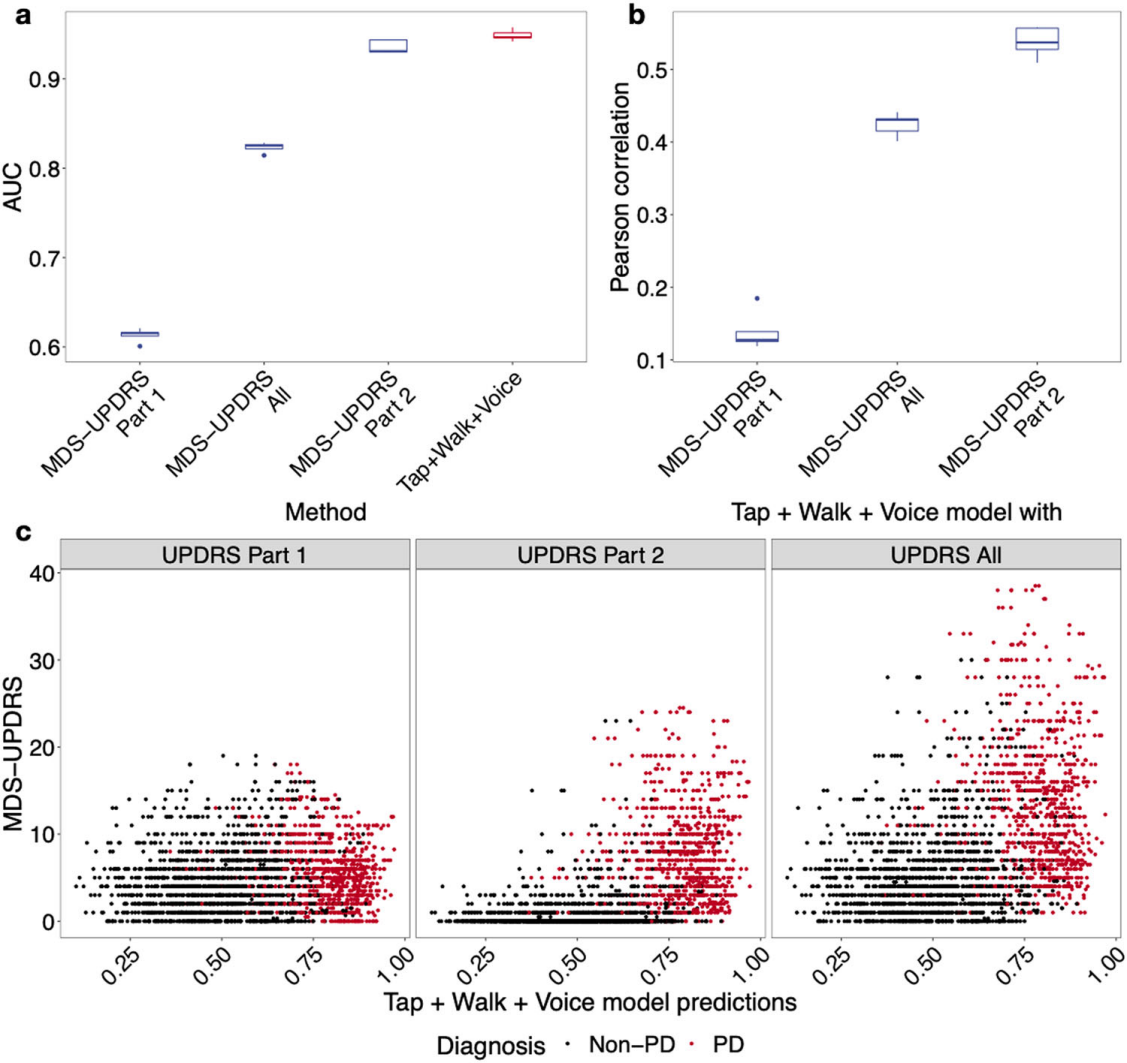
Correlation with patient self-reports. **a** AUCs of evaluating self-reported MDS-UPDRS against diagnosis and deep learning models against diagnosis. **b** Pearson correlations between self-reported MDS-UPDRS scores and deep learning-based predictions. **c** Visualization of the relationship between the MDS-UPDRS and the prediction values.

We further compared the performance of the prediction models against UPDRS (The Unified Parkinson’s Disease Rating Scale) patient self-reports, when they are available. The AUC of the combined deep learning model on these shared individuals is 0.9486. UPDRS directly evaluated on the binary label achieved an AUC of 0.8232, and UPDRS part 2 (motor experience of daily living) achieved an AUC of 0.9356 (Fig. 6a, Supplementary Table 5). This result suggests that the digital biomarkers can make more accurate predictions than patient self-reports. Additionally, prediction values from the combined deep learning model showed strong correlation with UPDRS scores and with UPDRS part 2 scores (0.4240 and 0.5378, respectively, Fig. 6b, c, Supplementary Fig. 5, Supplementary Table 6). This result supports the clinical relevance and utility of the deep learning model.

## Discussion

In this study, we proved the ability of finger-tapping positions on identifying the PwPs and the reported performances, an average AUC of 0.935, support that digital biomarkers for diagnosing PD can be developed beyond gross motor skills such as gait/rest, the primary focus of prior literature^33–35^. We also identified a digital biomarker approach that successfully predicts PwP status with even higher accuracy. This digital biomarker approach is based on integrative measurements of fine-motor skills and gross motor skills. The combined model achieved an average AUC of 0.944, a superior performance compared to patient self-reports. Compared to previous studies, our work has the largest dataset^4^, and presents the ability of deep learning techniques to retrieve a high accuracy on predicting the PD diagnosis^13^. Our movement data model also has a higher performance in AUC than the previous works using traditional machine learning techniques^11,12,36,37^.

However, lacking the information about how the diagnoses are determined, and the reliability of the self-reported conditions, our model can be influenced by the biases from different diagnostic criteria and the false-positive self-identifications. Besides, our models still have two potential limitations. First, the number of records per patient is different and the guideline allows the participant to submit data at any time they want. These lead to potential biases in analysis. We select the maximum scores for each individual to maximize the PD detection, but it may impair the classification for the non-PDs. Second, we include the data with abnormal tapping durations and tapping times. Although only less than 0.1% of the data have these abnormalities, it is possible for the model to learn the biases from them.

PD is characterized by motor abnormalities, specifically tremor and bradykinesia, with an impaired performance of fine-motor skills as a common early manifestation of PD. Micrographia, for example, is often present before the development of overt changes in gait, posture, or voice. Subtle changes in motor performance likely precede the emergence of overt PD^38^. Although the traditional motor-based diagnosis of PD, like the UPDRS, has already achieved a high accuracy, an easily accessible and robust method differentiating PwP from controls would be useful for screening the population, potentially including identification of prodromal PD. A complete MDS-UPDRS test requires the participant to fill a 33-page survey^39^, which is time consuming and hard especially for a PD patient with motor disorder. Our work provides a possible solution for the convenient, in-home PD assessment and progression follow-up. It also may provide useful biomarkers for the evaluation of interventions.

These methods might also be useful for evaluating more advanced PD subjects. Identification of digital biomarkers independent of walking/rest data is a potentially useful approach for evaluating important motor functions in more advanced PD patients. As PD progresses, patients often lose their ability to think and reason, along with walking, but will maintain the ability to carry out tapping tests for much longer.

In summary, our results indicate an integrative digital biomarker approach with several potential applications for PD detection and monitoring of disease activities. Our study also emphasizes the advantages of utilizing different types of biomarkers. Previous studies have indicated the relationships between the PD and the other modalities like the rapid eye movement (REM) sleep behavior disorder (RBD)^40^, the genotypes^41^, and their abilities on PD predictions^42,43^. Combining the digital biomarkers from these sources may further improve the accuracy of the models^4^, and may help distinguish PD with other types of tremulous movement disorders like SWEDD (Subjects Without Evidence of Dopaminergic Deficit)^44–46^. Possible future work also include a collection of long-term follow-up data to evaluate the model ability of predicting PD before diagnosis, and a collection of other movement disorders data like the cerebellar ataxia^47^, and the Alzheimer’s disease-associated movement disorders^48^, for distinguishing PD from them. Our work can easily transfer to these classification tasks with the transfer learning strategy^49^.

## Methods

### Ethics

The study data was downloaded from the mPower portal ([https://www.synapse.org/#!Synapse:syn5511439/tables/]). mPower study participants have agreed with secondary analysis of the data when signing on the APP. Additionally, all researchers that have access to the data in this study have obtained mPower permission.

### General mPower participant guideline

The data collection was opened to the individuals diagnosed with PD as well as anyone interested in participating as a control^2^. They must be 18 years of age or older, living in the United States, and comfortable reading and writing on an iPhone in English. Participants needed to take a quiz of the study aims, participants right and data sharing, and were required to complete an e-consent process and sign. They were also asked for an email for verification.

Participants can submit their records three times a day. Participants with PD were asked to finish the tests in three scenarios: (1) immediately before taking their medication; (2) after taking their medication (when they are feeling at their best); (3) at some other time. And those with non-PD can complete at any time.

### Deep learning architecture and training procedure

Similar neural network structures were built for accelerometer and coordinate data models except for channel lengths according to different sizes of input data: 2500 for the accelerometer model and 800 for the coordinate model. The network contains seven convolutional layers, seven max-pooling layers, and one fully connected layer activated by a sigmoid function for the output (Figs. 2a, 3a).

We performed cross-validation by partitioning the mPower samples into the training set (75%) and the testing set (25%) at the individual level with a random seed. Furthermore, half of the training samples were used to train the model and the other half were used in the validation process for hyperparameter tuning. During each training process, the binary cross-entropy *H*_*b*_(*p*) was applied as the loss function for selecting the best epoch, the parameters used in that epoch would then be stored for validation. The loss function is defined as:

1 $${H}_{b}(p)=-p\times {\log }(\hat{p})-(1-p)\times {\log }(1-\hat{p})$$

where *p* is the ground truth (1 or 0 in our case) and $\hat{p}$ is the prediction value. To evaluate the performances of different models, Area Under Receiver Operating Characteristic Curve (AUC) and Area Under Precision-Recall Curve (AUPRC) scores were calculated and compared. Notably, AUC would not be affected by the baseline accuracy (number of PD individuals/number of all individuals), thus was more stable in our imbalanced data^50^. Predicting most individuals as non-PD would retrieve a relatively high accuracy, while the AUC could be nearly random.

Adabound was an variant of the Adam optimizer employing dynamic bounds on the learning rate. It was claimed to have both a rapid training process and good generalization ability^51^. We implemented this algorithm in Theano and applied it to our coordinate data model, along with a series of hyperparameter tunings on input length, batch size, and learning rate. The model with Adabound optimizer, 800 input length, 8 batch size, and 1e−4 learning rate reached the best performance (Fig. 3b).

### Data normalization and augmentations

In order to avoid the potential data leakage, both the normalization and the augmentations are applied on each batch separately during the training process^23^.

Normalization methods in our experiments include a z-score normalization $({X}_{b}-\underline{{X}_{b}})/{{{{{\rm{std}}}}}}({X}_{b})$, a centering ${X}_{b}-\underline{{X}_{b}}$ (*X*_*b*_ is the data in a batch), and a boundary normalization using the bounds of the tapping areas. The boundary normalization is inspired by the fact that the resolutions of different devices may also contribute to the tapping position variances among the individuals. Calculating the relative positions by subtracting the bounds from the raw coordinates can eliminate the bias from devices.

The augmentations are scalings and rotations. To scale the time, we resize the lengths of the raw inputs with a coefficient randomly selected from (0.8, 1.2) using the OpenCV^52^. To scale the magnitude, we multiply the raw signals with coefficients randomly selected from (0.8, 1.2) on each channel. The rotations are implemented based on the quaternion rotation matrix^53^ for the 3-D (the accelerometer data) and the 2D (the coordinates data) objects. The alternative rotation ranges in our experiments are (0, 2π) and (−π/2, π/2).

### Pulling predictions of multiple records of a single individual and aggregation of the two models

Since mPower collected data from voluntary participants in an uncontrolled environment, each individual might perform one task multiple times and generate more than one record. We used the same pulling prediction strategy to deal with multiple records from the same individual as models we built on mPower walking data and voice data^15^. Since we applied a 5-fold cross-validation method during model training, we had five evaluation scores for the same record in each fold. We first averaged these five scores to one mean score and used this score in pulling. We tried two pulling methods—average pulling and maximum pulling. In average pulling, the mean of all the records from a single individual was calculated. In maximum pulling, the maximum evaluation score of an individual was picked. The final evaluation score was then used to predict the PwP status of that individual.

Since each tapping test collected both acceleration and tapping coordinate data, we were able to assemble the results of these two models to see if model performance improved. Since the pulling method improved the AUC score for each model, we used the pulled score for each individual during aggregation. The evaluation scores from the two models of the same individual were collected, which is similar to adding another feature in the single model. The mean of the two scores was calculated as the final score for that individual. We used this final score to predict the PwP status.

### Assembling gait and voice models on top of the tapping model

In order to compare among models built on different types of data, we identified the individuals with all types of these records. We used records from these individuals to train all four models (gait, voice, and tapping models) with the same split of 5-fold cross-validation. After comparing the performance of these single models, we tried to assemble gait and voice models on top of the best-performed tapping model (coordinate model). Similar strategies as aggregating two tapping models were used—the pulled score for each individual from the three models was collected together and averaged to a final score for the following evaluation.

### Statistics and reproducibility

Statistics were performed in the R Studio (R version 4.0.2). The p-values were calculated by the two-sided t-test on the AUCs from the 5-fold cross-validations. The models were constructed using Theano (version 1.0.2) and the Lasagne (version 0.2.dev1) in Python (version 2.7.5). More information about the environment and implementation details can be found in https://github.com/GuanLab/PDTap.

### Reporting summary

Further information on research design is available in the Nature Research Reporting Summary linked to this article.

## Supplementary information

Transparent Peer Review File

Supplementary Information

Description of Additional Supplementary Files

Supplementary Data

Reporting Summary

## Data Availability

The datasets used in our works are available through the mPower Public Researcher Portal (https://www.synapse.org/mpower). Researchers who are interested in accessing these data should follow the mPower Data Governance (https://github.com/Sage-Bionetworks/mPower-sdata) (Bot et al. 2016). All the data behind the figures are available in the zip file of the Supplementary Data.

## Code availability

Code is available at https://github.com/GuanLab/PDTap.
