# Supplementary material for: Heterogeneous digital biomarker integration out-performs patient self-reports in predicting Parkinson’s disease
Source: Commun Biol. 2022 Jan 17;5:58. doi: 10.1038/s42003-022-03002-x (PMC8763910; doi:10.1038/s42003-022-03002-x)

Supplementary Table 1. Performance comparison of models with accelerometer data.

Pool on individuals	Normalization	Augmentation	Mean AUC	Median AUC
**Experiments on the augmentation and normalization**				
NULL	NULL	NULL	0.7728	0.7767
Average	NULL	NULL	0.8549	0.8509
Maximum	NULL	NULL	0.8821	0.8834
NULL	Z-score	NULL	0.7944	0.8004
Average	Z-score	NULL	0.8795	0.8787
Maximum	Z-score	NULL	0.9045	0.9033
NULL	Z-score	Quaternion Rotation	0.8151	0.8218
Average	Z-score	Quaternion Rotation	0.8886	0.8893
Maximum	Z-score	Quaternion Rotation	0.9109	0.9095
NULL	Z-score	Scaled Magnitude<br>Quaternion Rotation	0.8171	0.8236
Average	Z-score	Scaled Magnitude<br>Quaternion Rotation	0.8887	0.8890
Maximum	Z-score	Scaled Magnitude<br>Quaternion Rotation	0.9111	0.9103
NULL	Z-score	Scaled Magnitude<br>Scaled Time<br>Quaternion Rotation	0.8340	0.8374
Average	Z-score	Scaled Magnitude<br>Scaled Time<br>Quaternion Rotation	0.8972	0.8970
Maximum	Z-score	Scaled Magnitude<br>Scaled Time<br>Quaternion	**0.9174**	**0.9161**

Rotation

Supplementary Table 2. Performance comparison for the models on the raw coordinate data and timestamps

Timestamp	Augmentation	Normalization	Network Settings	Mean AUC	Median AUC
**Experiments on the raw data**					
NULL	NULL	NULL	Input length: 2500<br>Batch size: 4<br>Optimizer: Adam<br>Learning rate: 5e-5	0.9279	0.9282
NULL	NULL	NULL	Input length: 800<br>Batch size: 4<br>Optimizer: Adam<br>Learning rate: 5e-5	0.9294	0.9267
RAW	NULL	NULL	Input length: 2500<br>Batch size: 4<br>Optimizer: Adam<br>Learning rate: 5e-5	0.9270	0.9268
RAW	NULL	NULL	Input length: 800<br>Batch size: 4<br>Optimizer: Adam<br>Learning rate: 5e-5	0.9279	0.9266
NULL	NULL	NULL	Input length: 800<br>Batch size: 8<br>Optimizer: Adam<br>Learning rate: 1e-4	0.9291	0.9289
NULL	NULL	NULL	Input length: 800<br>Batch size: 8<br>Optimizer: Adabound<br>Learning rate: 1e-4	**0.931**	**0.93**

Supplementary Table 3. Performance comparison for normalization methods for coordinate data

Timestamp	Augmentation	Normalization	Network Settings	Mean AUC	Median AUC
**Experiments on the augmentation and normalization**					
NULL	NULL	Centering	Input length: 800<br>Batch size: 4<br>Optimizer: Adam<br>Learning rate: 5e-5	0.9286	0.931
NULL	NULL	Centering	Input length: 800<br>Batch size: 8<br>Optimizer: Adam<br>Learning rate: 1e-4	0.9305	0.9336
NULL	NULL	Centering	Input length: 800<br>Batch size: 8<br>Optimizer: Adabound<br>Learning rate: 1e-4	0.9343	0.9328
Centering	NULL	Centering	Input length: 800<br>Batch size: 8<br>Optimizer: Adabound<br>Learning rate: 1e-4	**0.9352**	**0.9336**
NULL	NULL	Bound	Input length: 800<br>Batch size: 4<br>Optimizer: Adam<br>Learning rate: 5e-5	0.9176	0.9159
Centering	NULL	Centering	Input length: 800<br>Batch size: 4<br>Optimizer: Adam<br>Learning rate: 5e-5	0.9303	0.9321
Centering	NULL	Bound	Input length: 800<br>Batch size: 4<br>Optimizer: Adam<br>Learning rate: 5e-5	0.9148	0.9129

Supplementary Table 4. Performance comparison on augmentation methods for coordinate data

Timestamp	Augmentation	Normalization	Network Settings	Mean AUC	Median AUC
**Experiments on the augmentation**					
NULL	2D-rotation (-90° to 90°) + Time scaling	NULL	Input length: 800<br>Batch size: 4<br>Optimizer: Adam<br>Learning rate: 5e-5	0.9141	0.9191
NULL	2D-rotation (360°) + Time scaling	NULL	Input length: 800<br>Batch size: 4<br>Optimizer: Adam<br>Learning rate: 5e-5	0.9123	0.9149
NULL	2D-rotation (-90° to 90°) + Time scaling	Centering	Input length: 800<br>Batch size: 8<br>Optimizer: Adabound<br>Learning rate: 1e-4	0.9248	0.9278

Supplementary Table 5. Comparisons between the AUCs from using different parts of MDS-UPDRS and our methods to predict PD.

Model	Fold	Patient #	MDS-UPDRS All	MDS-UPDRS Part 1	MDS-UPDRS Part 2	Our Model
Tap + Walk + Voice	1	506	0.8264	0.6161	0.9293	0.9418
Tap + Walk + Voice	2	491	0.8144	0.6154	0.9310	0.9463
Tap + Walk + Voice	3	508	0.8283	0.6208	0.9435	0.9513
Tap + Walk + Voice	4	528	0.8252	0.6123	0.9302	0.9461
Tap + Walk + Voice	5	507	0.8217	0.6008	0.9440	0.9576
Average			0.8232	0.6131	0.9356	**0.9486**
Accelerometer	1	946	0.7904	0.5621	0.9094	0.9183
Accelerometer	2	952	0.7942	0.5841	0.9096	0.9025
Accelerometer	3	944	0.8168	0.6041	0.9212	0.9152
Accelerometer	4	995	0.8078	0.5864	0.9177	0.9162
Accelerometer	5	949	0.8224	0.6234	0.9190	0.9176
Average			0.8063	0.5920	**0.9154**	0.9139
Coordinates	1	508	0.8285	0.6053	0.9266	0.9157
Coordinates	2	488	0.8108	0.6118	0.9025	0.9385
Coordinates	3	490	0.8138	0.5864	0.9155	0.9417
Coordinates	4	479	0.8007	0.6036	0.9101	0.9294
Coordinates	5	477	0.8098	0.6116	0.9097	0.9336
Average			0.8127	0.6037	0.9129	**0.9318**

Supplementary Table 6. Pearson correlations between the inferences from our methods and the MDS-UPDRS scores

Model	Fold	Patient #	MDS-UPDRS All	MDS-UPDRS Part 1	MDS-UPDRS Part 2
Tap + Walk + Voice	1	506	0.4152	0.1276	0.5275
Tap + Walk + Voice	2	491	0.4012	0.1388	0.5093
Tap + Walk + Voice	3	508	0.4318	0.1252	0.5569
Tap + Walk + Voice	4	528	0.4412	0.1846	0.5371
Tap + Walk + Voice	5	507	0.4307	0.1188	0.5579
Average			0.4240	0.1390	0.5378
Coordinates	1	508	0.3564	0.0760	0.4869
Coordinates	2	488	0.3875	0.0897	0.5135
Coordinates	3	490	0.3908	0.0932	0.5144
Coordinates	4	479	0.4072	0.1243	0.5130
Coordinates	5	477	0.3905	0.1132	0.5097
Average			0.3865	0.0993	0.5075
Accelerometer	1	946	0.3381	0.0428	0.4684
Accelerometer	2	952	0.3467	0.0912	0.4500
Accelerometer	3	944	0.3655	0.0796	0.4832
Accelerometer	4	995	0.3515	0.0645	0.4709
Accelerometer	5	949	0.4125	0.1405	0.5085
Average			0.3628	0.0837	0.4762

Supplementary Figure 1. Results of models with improved performances. (A) are the experiments under "Raw Data". In Addition to the results listed in Figure 4, we also have the experiments on the padding lengths (2500 and 800), batch sizes (4 and 8), and the optimizer learning rates (1e-4 and 5e-5). (B) are the experiments under the "Centering" normalization. Note that we also compare these models with the previous best "plain" model (only use raw coordinates data).

a

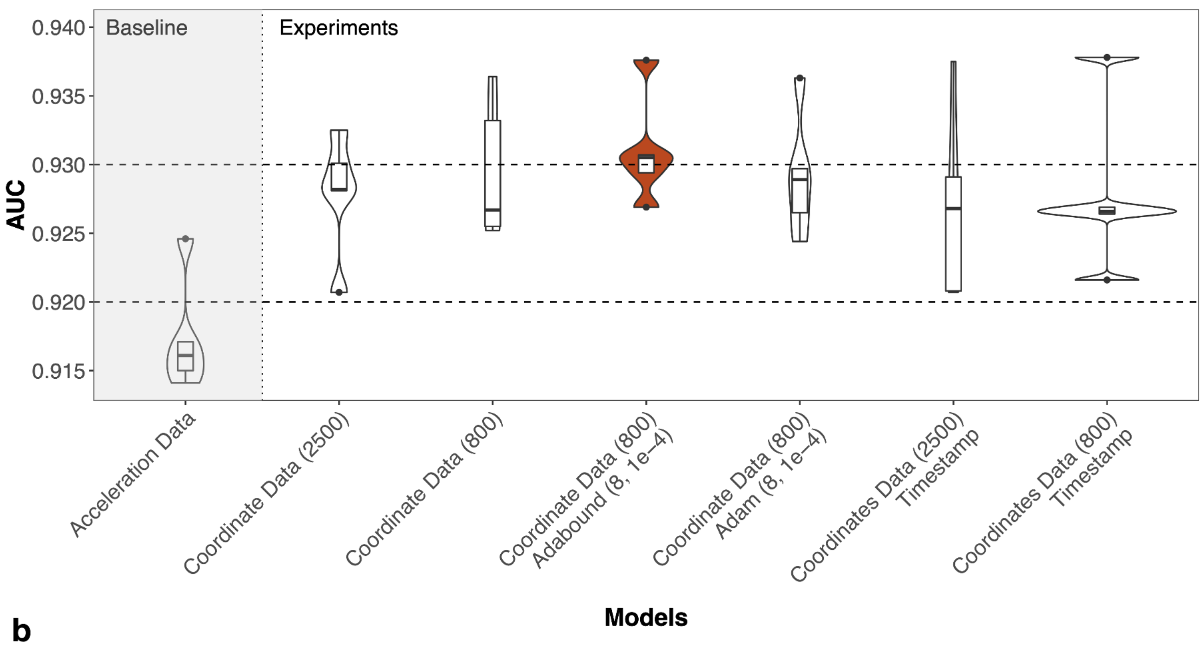

b

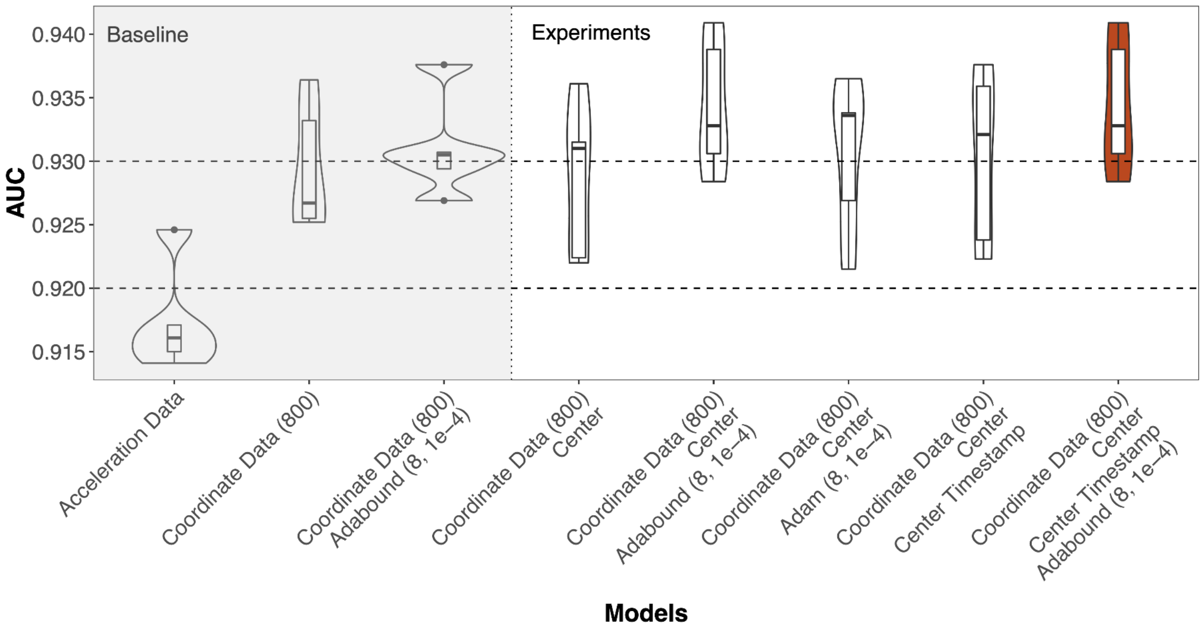

Supplementary Figure 2. Results of models with impaired performances. (A) show the experiments of using button position for normalization, and (B) are the experiments of augmentations. Adabound shows relatively stable performance. Compared to the baseline performances, all of these models have lower scores.

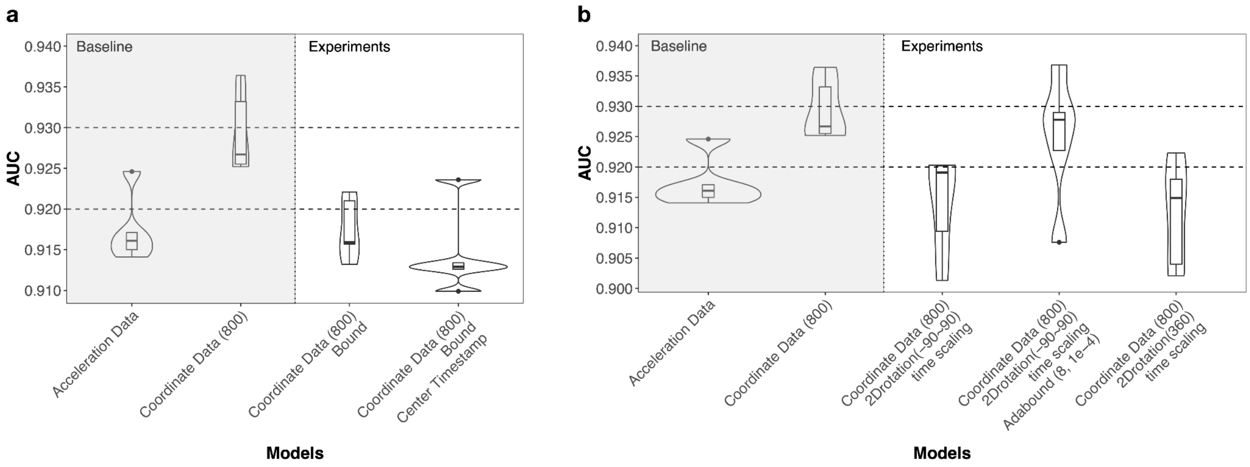

Supplementary Figure 3. Prediction performances with different augmentation methods on voice records on record level (before the pull, upper panel) and individual level (after max pull, lower panel).

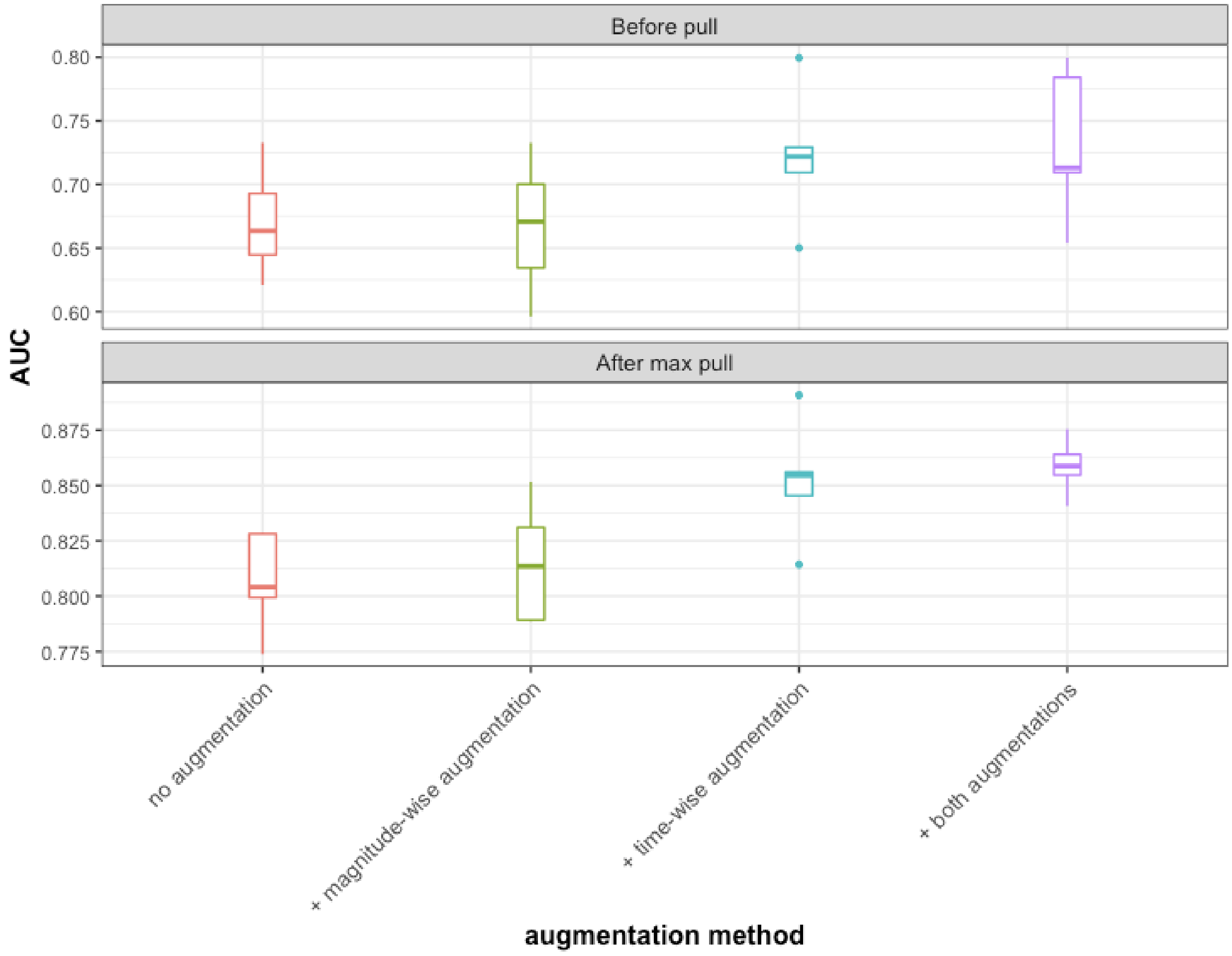

Supplementary Figure 4. The relationships between the prediction values of the three models and age/smoke year.

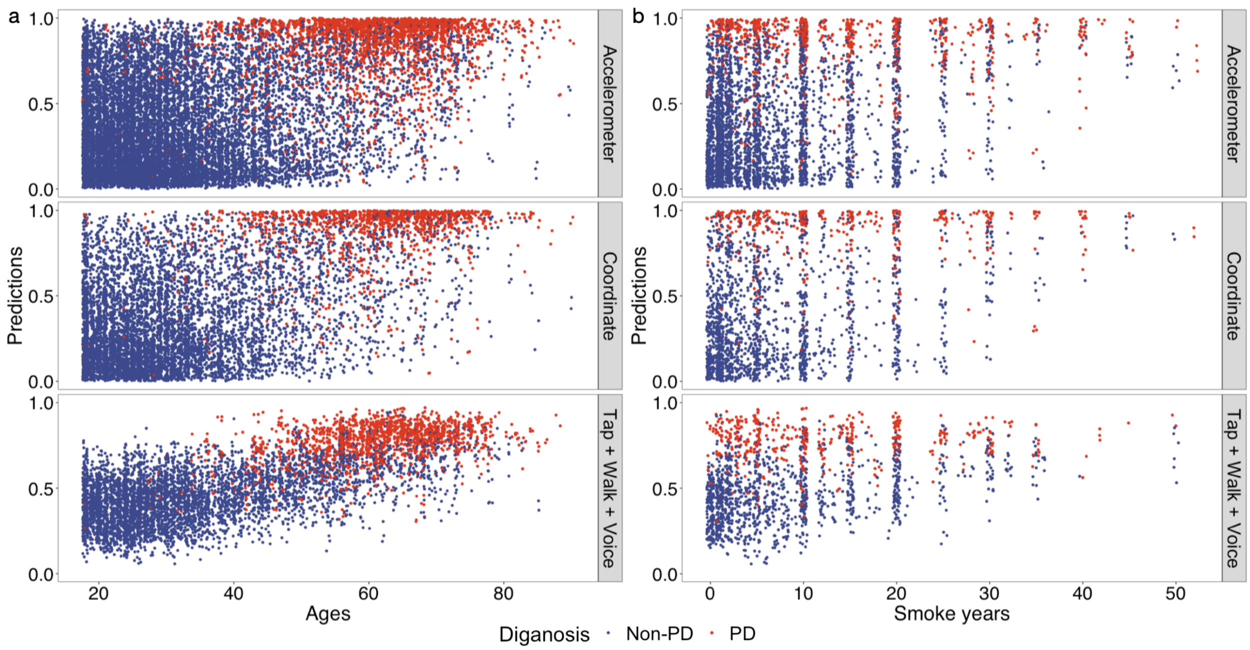

Supplementary Figure 5. Correlations between MDS-UPDRS scores and the inferences from our methods visualized in data points. The blue lines are the linear regression fits on these two types of data. In this plot matrix, each column represents a fold in the cross-validation, and each row represents the summation of a part of the MDS-UPDRS (Part 1, Part 2, and all).

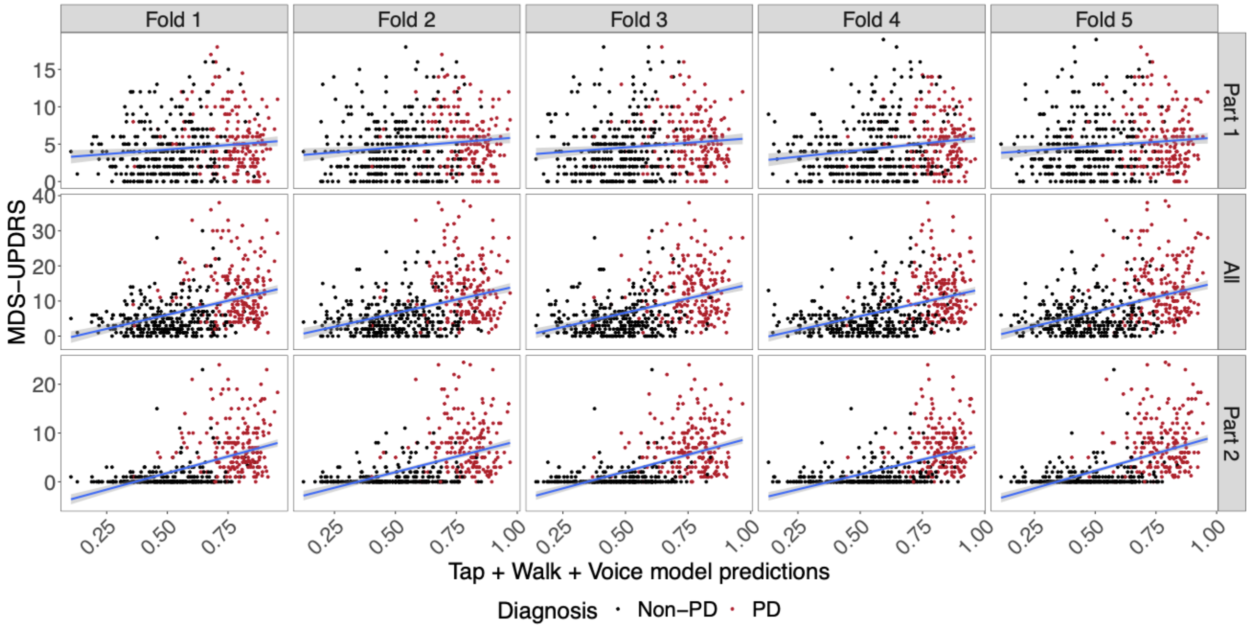

Supplement: Supplementary file 2 — Supplementary Information [file 42003_2022_3002_MOESM2_ESM.pdf]
