# Supplementary material for: Heterogeneous digital biomarker integration out-performs patient self-reports in predicting Parkinson’s disease
Source: Commun Biol. 2022 Jan 17;5:58. doi: 10.1038/s42003-022-03002-x (PMC8763910; doi:10.1038/s42003-022-03002-x)
Supplement: Supplementary file 3 — Description of Additional Supplementary Files [file 42003_2022_3002_MOESM3_ESM.pdf]

Description of Additional Supplementary Files

File name: Supplementary Data
Description: The source data for generating the figures from Figure 2 to Figure 6.
