# Supplementary material for: Heterogeneous digital biomarker integration out-performs patient self-reports in predicting Parkinson’s disease
Source: Commun Biol. 2022 Jan 17;5:58. doi: 10.1038/s42003-022-03002-x (PMC8763910; doi:10.1038/s42003-022-03002-x)
Supplement: Supplementary file 5 — Reporting Summary [file 42003_2022_3002_MOESM5_ESM.pdf]

nature portfolio

Corresponding author(s): Yuanfang Guan

Last updated by author(s): 11/29/2021

Reporting Summary

Nature Portfolio wishes to improve the reproducibility of the work that we publish. This form provides structure for consistency and transparency in reporting. For further information on Nature Portfolio policies, see our Editorial Policies and the Editorial Policy Checklist.

Statistics

For all statistical analyses, confirm that the following items are present in the figure legend, table legend, main text, or Methods section.

n/a	Confirmed	
☐	☒	The exact sample size (*n*) for each experimental group/condition, given as a discrete number and unit of measurement
☐	☒	A statement on whether measurements were taken from distinct samples or whether the same sample was measured repeatedly
☐	☒	The statistical test(s) used AND whether they are one- or two-sided<br>*Only common tests should be described solely by name; describe more complex techniques in the Methods section.*
☐	☒	A description of all covariates tested
☐	☒	A description of any assumptions or corrections, such as tests of normality and adjustment for multiple comparisons
☐	☒	A full description of the statistical parameters including central tendency (e.g. means) or other basic estimates (e.g. regression coefficient) AND variation (e.g. standard deviation) or associated estimates of uncertainty (e.g. confidence intervals)
☐	☒	For null hypothesis testing, the test statistic (e.g. *F*, *t*, *r*) with confidence intervals, effect sizes, degrees of freedom and *P* value noted<br>*Give P values as exact values whenever suitable.*
☐	☒	For Bayesian analysis, information on the choice of priors and Markov chain Monte Carlo settings
☐	☒	For hierarchical and complex designs, identification of the appropriate level for tests and full reporting of outcomes
☐	☒	Estimates of effect sizes (e.g. Cohen's *d*, Pearson's *r*), indicating how they were calculated

Our web collection on statistics for biologists contains articles on many of the points above.

Software and code

Policy information about availability of computer code

Data collection	Raw data were collected by the work: "Bot, B. M. et al. The mPower study, Parkinson disease mobile data collected using ResearchKit. Sci Data 3, 160011 (2016)". We downloaded from the mPower Public Researcher Portal: https://www.synapse.org/#!Synapse:syn5511439/tables/."
Data analysis	The data analysis and modeling codes were written in Python 2.7.5, using the packages Theano 1.0.2 and Lasagne 0.2.dev1. The visualizations and statistical analysis were implemented in R 4.0.2.

For manuscripts utilizing custom algorithms or software that are central to the research but not yet described in published literature, software must be made available to editors and reviewers. We strongly encourage code deposition in a community repository (e.g. GitHub). See the Nature Portfolio guidelines for submitting code & software for further information.

Data

Policy information about availability of data

All manuscripts must include a data availability statement. This statement should provide the following information, where applicable:

- Accession codes, unique identifiers, or web links for publicly available datasets
- A description of any restrictions on data availability
- For clinical datasets or third party data, please ensure that the statement adheres to our policy

The datasets used in our works are available through the mPower Public Researcher Portal (https://www.synapse.org/mpower). Researchers who are interested in accessing these data should follow the mPower Data Governance (https://github.com/Sage-Bionetworks/mPower-sdata) (Bot et al. 2016). All the data behind the figures are available in the zip file of the Supplementary Data.

Field-specific reporting

Please select the one below that is the best fit for your research. If you are not sure, read the appropriate sections before making your selection.

☒ Life sciences ☐ Behavioural & social sciences ☐ Ecological, evolutionary & environmental sciences

For a reference copy of the document with all sections, see nature.com/documents/nr-reporting-summary-flat.pdf

Life sciences study design

All studies must disclose on these points even when the disclosure is negative.

Sample size	8003 individuals with 78879 records
Data exclusions	Records without the self-reported diagnosis were excluded.
Replication	5-fold cross-validation for evaluating the model performances
Randomization	In each fold of the cross-validation, we randomly selected 50% of the individuals for training, 25% for validation and 25% for testing.
Blinding	The models were blind to the test data during the training and tuning. Hyper-parameters were determined by the performances on the validation sets.

Reporting for specific materials, systems and methods

We require information from authors about some types of materials, experimental systems and methods used in many studies. Here, indicate whether each material, system or method listed is relevant to your study. If you are not sure if a list item applies to your research, read the appropriate section before selecting a response.

Materials & experimental systems

n/a	Involved in the study	
☒	☐	Antibodies
☒	☐	Eukaryotic cell lines
☒	☐	Palaeontology and archaeology
☒	☐	Animals and other organisms
☒	☐	Human research participants
☒	☐	Clinical data
☒	☐	Dual use research of concern

Methods

n/a	Involved in the study	
☒	☐	ChIP-seq
☒	☐	Flow cytometry
☒	☐	MRI-based neuroimaging
